# Evaluation of different defect-inspection setups for injection molding parts based on the deep learning method

**DOI:** 10.1038/s41598-026-52635-z

**Published:** 2026-08-05

**Authors:** Enrico Bovo, Xi Vincent Wang, Giovanni Lucchetta, Lihui Wang

**Affiliations:** 1 Department of Production Engineering, KTH Royal Institute of Technology, Stockholm, 10044 Sweden; 2 Department of Industrial Engineering, University of Padua, Via Venezia 1, Padua, 35131 Italy

**Keywords:** Injection Molding, Surface Quality, Automated Optical Inspection, Defect Detection, Deep Learning, Engineering, Mathematics and computing

## Abstract

In the injection molding industry, the shift toward small-batch production has led to a greater variety of products and smaller batch sizes, necessitating frequent mold changes and efficient quality control, which still largely relies on human operators. This study proposes a comprehensive methodology for evaluating and comparing deep learning-based automatic optical inspection (AOI) strategies to detect complex surface defects in injection-molded parts. Three inspection setups were assessed: static frontal imaging, belt conveyor inspection, and robotic-assisted inspection. The findings reveal clear differences in defect detection capabilities among the methods, with the robotic-assisted approach demonstrating superior performance, achieving higher defect detection accuracy due to its flexibility in optimizing camera angles and positions. The proposed methodology serves as a workflow to systematically evaluate and optimize inspection setups across different parameters, enabling informed decisions about AOI systems design. This research contributes to narrowing the gap between the development of advanced detection algorithms and their industrial application, offering insights into the strategic implementation of AI technologies in quality control processes and enhancing the automatic detection of challenging defects.

## Introduction

Product customization is an increasing demand in most industries. Driven by lean production principles, manufacturing is shifting towards product personalization, resulting in a large product variety and small batch sizes^1^. In Injection Molding (IM) plastic parts, this leads to more frequent mold changes and machine startups. IM is a complex process with many interdependent variables, making quality prediction difficult^2^. Additionally, many injection-molded components are designed with a focus on appearance. The surface appearance primarily governs the perceived quality, often resulting in the dismissal of products with visual imperfections^3^. Hence, the quality control phase is essential to verify that products are manufactured in accordance with the specifications and requirements established during the initial stages of product development^4^. At each machine startup, input parameters are optimized to achieve high-quality products efficiently and economically^5^. The typical approach often relies on experience- and intuition-based procedures. Machine operators inspect the molded part and adjust the process parameters until the component’s quality meets the requirements. However, defective features on the surface of the plastic can vary significantly. Under these circumstances, extensive training is necessary for operators to detect defective products^6^, while finding experienced personnel is becoming more challenging. Moreover, human subjectivity highly impacts detection results, with the accuracy of quality assurance significantly depending on the inspector in charge^7^. Quality control strategies without human intervention thus remain among the most desired features in injection molding^8^, and more elaborate methods are required to ensure stable and reliable defect detection results^9^.

Recently, deep learning (DL) has become important for automated quality monitoring and has shown robustness to background, lighting, color, shape, size, and intensity when detecting patterns in images. This is especially useful for detecting complex surface defects in manufactured goods^10^. Wang et al.^11^ improved the YOLO v7 algorithm for detecting defects on steel surfaces, Zhang et al.^12^ developed a DL-based method for solar cell defect detection to handle complex image backgrounds, and Liu et al.^13^ enhanced the YOLO v4 algorithm for greater accuracy in fabric defect detection. DL-based computer vision systems have recently been adopted in the IM field for defect detection. Hu et al. improved a VGG16 network to classify different IM failures^14^, and Ha et al. developed a defect detection system for IM using data augmentation and edge computing^15^. In the works of Lee et al. and Liu et al., the authors proved that high defect detection performance can be achieved using pre-trained models, thus transferring knowledge from generic vision tasks to industrial inspection of plastic components^16,17^. The studies with promising detection results noted that the types of defects analyzed were limited to specific anomalies such as short shots, flashes, cracks, crazes, black spots, burrs, and pits. However, IM parts are often affected by sink marks, silver streaks, weld lines, and other defects that might be more difficult to detect, as defective areas become visible only when observed from different orientations^18^. IM machine operators manipulate the part to ensure that the products meet the required standards. More specifically, they intuitively position the surface of a part for the best specular light reflection to get the best contrast of surface disturbances^19^. The methodology adopted to perform visual inspection plays a crucial role in developing an effective automated optical inspection (AOI) system for IM parts. Key considerations include camera positioning. Typically, IM parts are demolded by a three-axis robot that positions the part in front of a static camera for quality control. Alternatively, ejected parts can be placed on a conveyor belt, where one or more cameras are strategically positioned to cover different inspection angles during transportation. In some cases, a single static frontal image of the component may be adequate for a comprehensive evaluation of product quality. On the other hand, video data can be more effective for inspecting the part from a wider range of angles, thereby enhancing the visibility of hard-to-spot defects. The number of frames analyzed significantly impacts the defect detection rate, as capturing multiple images of the same part increases the likelihood of identifying potential anomalies. Consequently, data redundancy can be used to ensure consistent detection results and enhance the reliability of the inspection system^20^. Lighting conditions also influence the success rate of detection, as proper illumination can help enhance the contrast of defective surface patterns^21^.

While many research works address well-known challenges in the application of DL-based AOI systems, such as imbalanced and small datasets, annotation efforts, noisy labels, and lack of transparency in black-box networks^22^, through techniques like Data Augmentation^23–25^, Transfer Learning^26–28^, Semi- and Un-supervised Learning^29–31^ among others, fewer studies have thoroughly investigated the effects of the inspection system setup on the defect detection success rate. Additionally, while much of the research is focused on enhancing DL algorithms for specific applications^32–34^, there is a significant gap in developing a comprehensive methodology for the practical deployment of these technologies in real-world manufacturing environments. This leads to a lack of refined, application-oriented methods to apply DL techniques for specific use cases^35^. In the work of Zhang et al.^36^, the authors highlight the necessity for a rigorous protocol for the quantitative evaluation of cracked concrete surfaces. Despite the images being collected under various lighting conditions, the study focused more on the image processing pipeline rather than comparing detection results obtained from different setups. Similarly, a comparative study of different AOI systems using the same inspection algorithm was conducted by Huang et al.^37^, but this research developed and studied two highly specific fixtures instead of proposing a general evaluation methodology. Other common issues in the literature related to the application of DL methods in the manufacturing field concern the evaluation procedure of defect detection algorithms. Firstly, it is a common practice that multiple images of the same component are first acquired to create a unique dataset, which is then split into training, validation, and testing sets. Rauber et al.^38^ discuss a similar issue in the context of machine learning methods for fault diagnosis, where chunks of the same signal are used in both the training and testing phases, referring to this problem as the similarity bias problem. Secondly, the performance of object detection algorithms is usually evaluated using common metrics such as Accuracy, Recall, Intersection-over-Union, Average Precision (AP), and the mean Average Precision (mAP). In real manufacturing inspections, the same component undergoes multiple assessments as object detection algorithms analyze multiple frames. Consequently, defects may only be detected in certain frames, while at the same time, only a fraction of the total number of frames might be detected per frame. The total number of defects detected, as well as the total number of true positives and false positives per each part, can thus represent more useful metrics to compare and evaluate different DL-based AOI systems.

In this study, a methodology for the evaluation and comparison of different DL-based AOI strategies is proposed. The research focuses on the visual inspection of hard-to-spot surface defects in injection-molded (IM) parts. Three distinct strategies are considered for developing the visual inspection system: static frontal images of the part, belt conveyor inspection mode, and inspection using a collaborative robot. For each technique, a data collection phase is conducted to acquire videos of both normal (good) and defective parts. A DL algorithm is then trained on the collected data and tested on videos of new testing samples. Custom metrics are employed to evaluate the performance of each inspection strategy. Key aspects of the different methodologies are discussed, including the use of videos over images, the rate of sampling and inference, and the various setups for data collection and visual inspection.

## Contributions

The main contribution of this work is the proposal of an inspection strategy to effectively detect hard-to-spot surface defects in injection-molded (IM) parts. A reproducible evaluation protocol for AOI is presented that makes part-level decisions from multi-frame sequences under declared aggregation rules and operating thresholds. Three industrially realistic setups (static rig, conveyor, robot-assisted) are compared under identical illumination using mode-specific YOLOv8s models trained under the same architecture and hyperparameter settings, isolating the effect of camera/part kinematics. Part-level metrics and precision–recall analysis are formalized to reflect the cost asymmetry between false negatives and false positives in production. A trajectory time-reallocation procedure for robot inspection is detailed, increasing correctly detected defective parts at fixed inspection time. Decisions are formed by explicit aggregation: for a sequence of *N* frames with per-frame matched detections *ndt* ​, a part is declared defective if:

$$\:{S}_{\tau\:}=\left|\left\{i:\:{ndt}_{i}\ge\:\tau\:\right\}\right|\ge\:K$$

Where $\:\tau\:$ and *K* are threshold values stated a priori and held constant across setups unless otherwise specified.

**Fig. 1:**
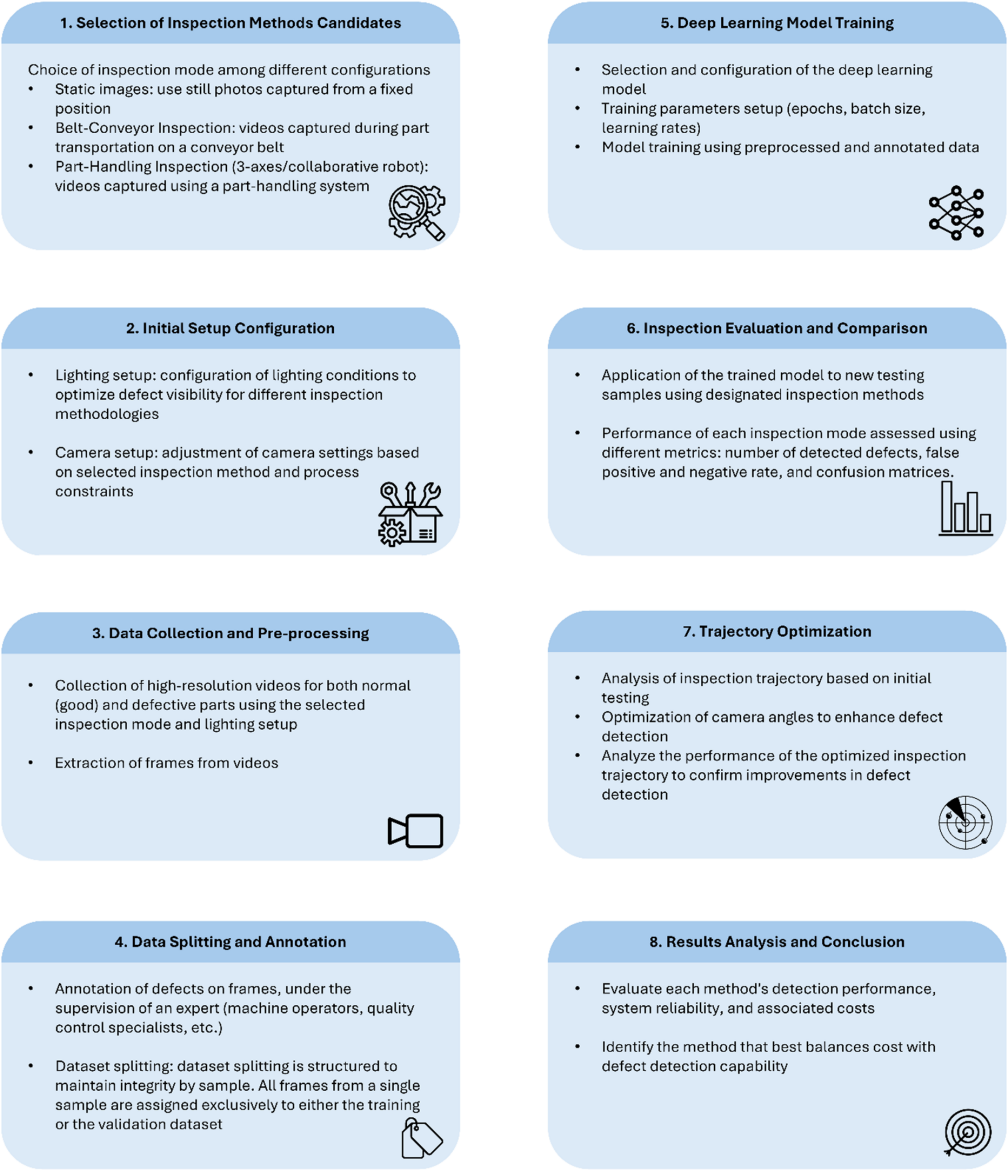
Flowchart of the proposed method for evaluating and comparing different AOI systems.

A flowchart of the proposed method is shown in Fig. 1.

Overall, this study addresses the primary limitations of previous research by introducing a general methodology to evaluate and compare different setups for implementing automated defect inspection systems in the IM manufacturing context, focusing on the practical application of state-of-the-art object detection models rather than developing novel algorithms. Unlike previous works in the field, this study addresses key aspects in the development of an AOI system by assigning different samples (manufactured parts) to either the training or validation dataset (to avoid the similarity bias problem) and adopting custom metrics during model evaluation that privilege the need for a highly reliable inspection system in real manufacturing applications.

## Materials and methods

### Inspection strategies

**Fig. 2:**
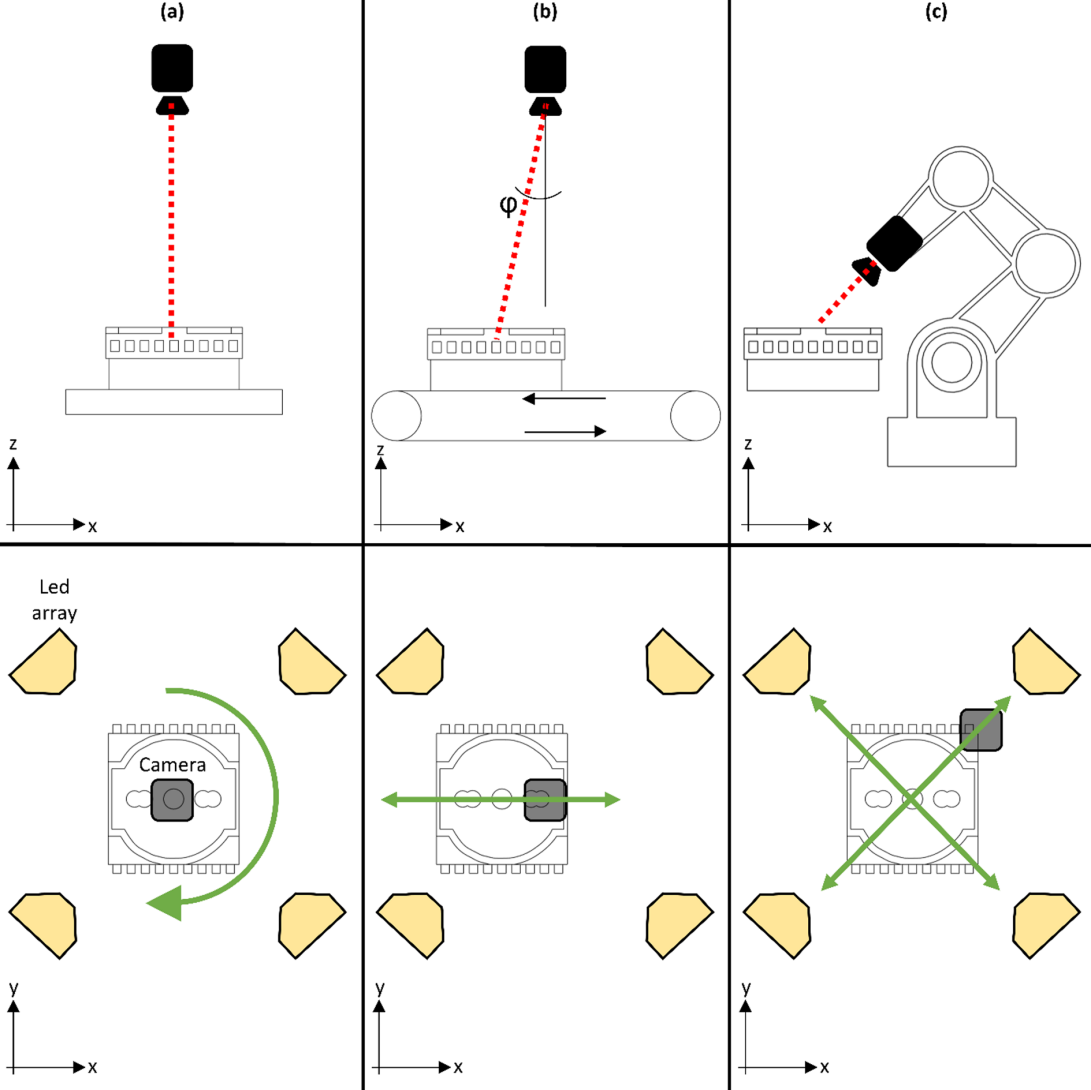
Three different inspection system configurations used for the experiments: frontal images (Mode I) (**a**), “belt-conveyor” inspection (Mode II) (**b**), and robot inspection (Mode III) (**c**). Top images: lateral view. Bottom images: top view. The green lines represent the motion of the part/camera for each configuration.

To investigate the effect of the AOI system on the success rate of defect detection, three different techniques were considered. A schematic of the three analyzed configurations is reported in Fig. 2.

The first technique (Fig. 2, a) consisted of the frontal frames acquisition of the part (Mode I). This method is equivalent to the very common inspection system used in the IM manufacturing field, where the demolded part is grabbed by a three-axis robot and presented in front of a static camera. The part is then rotated around the z-axis, which is perpendicular to the inspection plane. To perform the inspection, the part was placed on a small rotating table, enabling changes in light reflection at various rotation angles. The second method (Fig. 2, b), called belt conveyor inspection, simulates the inspection during part transportation on a belt conveyor (Mode II). It is very common for IM parts to be grabbed and placed over a belt conveyor before packaging. In this case, while the part does not rotate around the z-axis, an angle φ between the axis of the camera and the position of the part on the belt changes during transportation due to a translation in the x direction. Finally, the third technique (Fig. 2, c) leverages the use of a collaborative robot that holds the camera while the part remains stationary (Mode III) Although 3-axis robots are prevalent in the IM manufacturing field, a limited number of collaborative robots per plant may also be available. In such cases, a mobile inspection station can be equipped with one of these robots, enabling its transfer from one extrusion line to another- which is especially advantageous when automatic inspection is primarily required during startup phases. This inspection method allows for the widest range of inspection angles as the robot can move around the part, rotating the camera around the x, y, and z axes of the end effector. The selected inspection trajectory is depicted by the green lines at the bottom of the figure. The camera moved along a cross-shaped path while simultaneously varying the angle between the camera’s axis and the z-direction within a ± 30° range. The part was always placed randomly under the camera before inspection to avoid introducing bias from consistently favorable or unfavorable initial camera angles. For Modes I and III, this involved positioning the part at the center of the camera field with a random rotation angle around the z-axis, while For Mode II, the part was randomly positioned within the camera field, varying both the rotation angle as well as the x- and y-position. All three techniques allowed for changing the reflectance angle of the light over the surface of the part, thus enhancing defect visibility. The lighting setup was fixed. Four small LED arrays were positioned at the four corners of the inspection area and remained unchanged across all configurations.

### Equipment and data collection

The inspected IM part was the cover of an electrical socket, manufactured using black polycarbonate (Makrolon 2405, Covestro, Leverkusen, Germany). The most critical defects in this component are the so-called weld lines around the pins’ holes. These defects arise from the convergence of multiple fronts of melted material at a junction, resulting in a visible mark. Due to the black color and opacity of the part, these marks can become visible and invalidate the quality of the part. The formation of a weld line is unavoidable due to the nature of the process. However, the process conditions influence the intensity of the defect, with visibility ranging from highly visible to non-perceivable, determining if the part is classified as good. Therefore, inspecting the part is not straightforward, and different inspectors, or even the same inspector at different times, can give different inspection results.

The evaluated component is representative of a broad class of injection-molded parts: it exhibits weld-line defects on a matte polymer surface, together with common geometric features (curved shells, ribs, bosses) that induce similar shading patterns. These characteristics—shared by many consumer, automotive, and appliance housings—are likely to produce low-contrast surface irregularities that make defect detection challenging. For these reasons, the comparative methodology can be extended to additional parts with similar optical and morphological characteristics to evaluate alternative inspection setups.

**Fig. 3:**
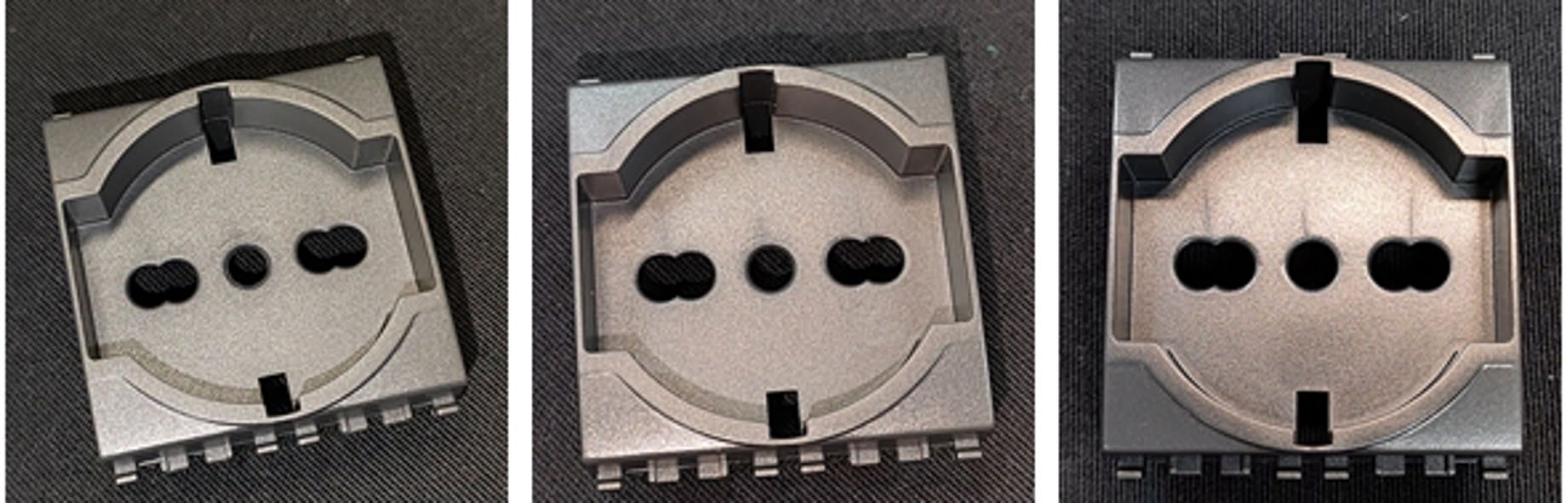
Weld-lines visibility for different samples. From left to right: not perceivable (good), slightly visible (defective), and highly visible (defective).

An example of a part with non-perceivable, slightly- and highly visible weld lines is reported in Fig. 3.

To train the object detection model, 81 samples were considered, 36 of which were normal (without visible defects) and the remaining 45 were defective. For each part, a 30-second inspection video was acquired using the three different techniques. To fairly evaluate the effect of the inspection method on the defect detection performance, the same camera settings and lighting conditions were used for all the experiments. An 8.9 MPx industrial camera was used to acquire high-resolution videos of the part, while four LED arrays were placed at the corners of the inspection table. The collaborative robot used for the experiments was a UR5 Universal Robot. To acquire the video of the part according to the first inspection mode, varying only the rotation angle around its symmetry axis, a small rotation table was used to rotate the part. The rotation speed was set so that a complete 360° rotation was performed in 30 s, while the camera remained stationary above the part. For what concerns the second inspection mode, the collaborative robot was used to simulate a realistic relative motion between the part and the camera during transportation over a belt conveyor. The last inspection mode fully utilized the collaborative robot’s capabilities, as the camera was moved around the part, rotating around the x, y, and z axes of the end effector. A “double cross” trajectory was defined using the command console of the UR5 Robot. A Python script was then used in conjunction with a simple object detection algorithm to open each video, sample the frames at fixed time intervals, detect the part, extract and resize the region of interest to a 1024 × 1024 p image to ensure uniform dimensions. For each video, the same number of frames was retrieved to build the training dataset. Overall, this resulted in approximately 800 labeled images per inspection mode (≈ 2400 images in total), providing a substantial amount of training data for learning defect appearance despite the limited number of physical parts. Since frames extracted from the same part are correlated, data splitting and performance evaluation are performed at the part level. The images were manually annotated under the supervision of an expert machine operator from the company using a custom annotation tool developed by the authors. The annotation app was developed in Python using the open-source library Streamlit, and utilized the drawable canvas API to annotate the images by creating bounding boxes around the defects. The dataset for each technique category was then split into training and validation sets in an 85 − 15% proportion.

All dataset splits were performed at the part level. Each physical part has a unique ID, and all frames extracted from the same part were kept within a single split to avoid leakage across training and testing. The 85–15% train/validation split was applied to the list of parts, and the corresponding frames inherited the part assignment. The test set consisted of 26 additional parts (16 defective, 10 normal) acquired separately, and the same 26 parts were inspected under all three modes.

### Annotation and inter-operator correspondence

All images were independently labeled twice by two injection-molding experts from the same company (Annotators A1 and A2) using shared guidelines. Labels consisted of axis-aligned bounding boxes around visible surface defects; annotators had no access to each other’s work. Inter-operator correspondence was summarized by micro-averaged F1-0.50 (IoU) using one-to-one matching on each image (pairs accepted if IoU ≥ 0.5; unmatched A1 boxes counted as FN, unmatched A2 boxes as FP).

A consensus ground-truth set was then derived as follows: (1) for each image, A1–A2 boxes were matched one-to-one with IoU ≥ 0.5; matched pairs were merged into a single box by averaging coordinates; (2) unmatched boxes were adjudicated and retained only if the frame was labeled “defect present” by at least one annotator and the box was visually confirmed during adjudication; otherwise they were flagged as low-confidence and excluded from training; (3) the merged and adjudicated boxes constituted the final consensus labels used in subsequent experiments.

### Deep Learning Algorithm

The DL algorithm used in this paper is YOLO-v8. YOLO-v8 is one of the most recent versions of the YOLO (You Only Look Once) models, a one-stage detector renowned for its accuracy and inference speed^39^. Given the size of each dataset and to contain the inference time, the “small” version YOLO-v8s was chosen. Once the model and training parameters were selected (reported in Table 1), the same algorithm was trained three times, once for each dataset. An early stop mechanism was also enabled to avoid overfitting during training.

**Table 1:** Object detection model training parameters.

Model	Task	Image size	Epochs	Early stopping	Batch size	Learning rate initial	Learning rate final
YOLO-V8s	Detection	1024 × 1024p	200	35	42	0.025	0.010

### Evaluation metrics and testing

**Fig. 4:**
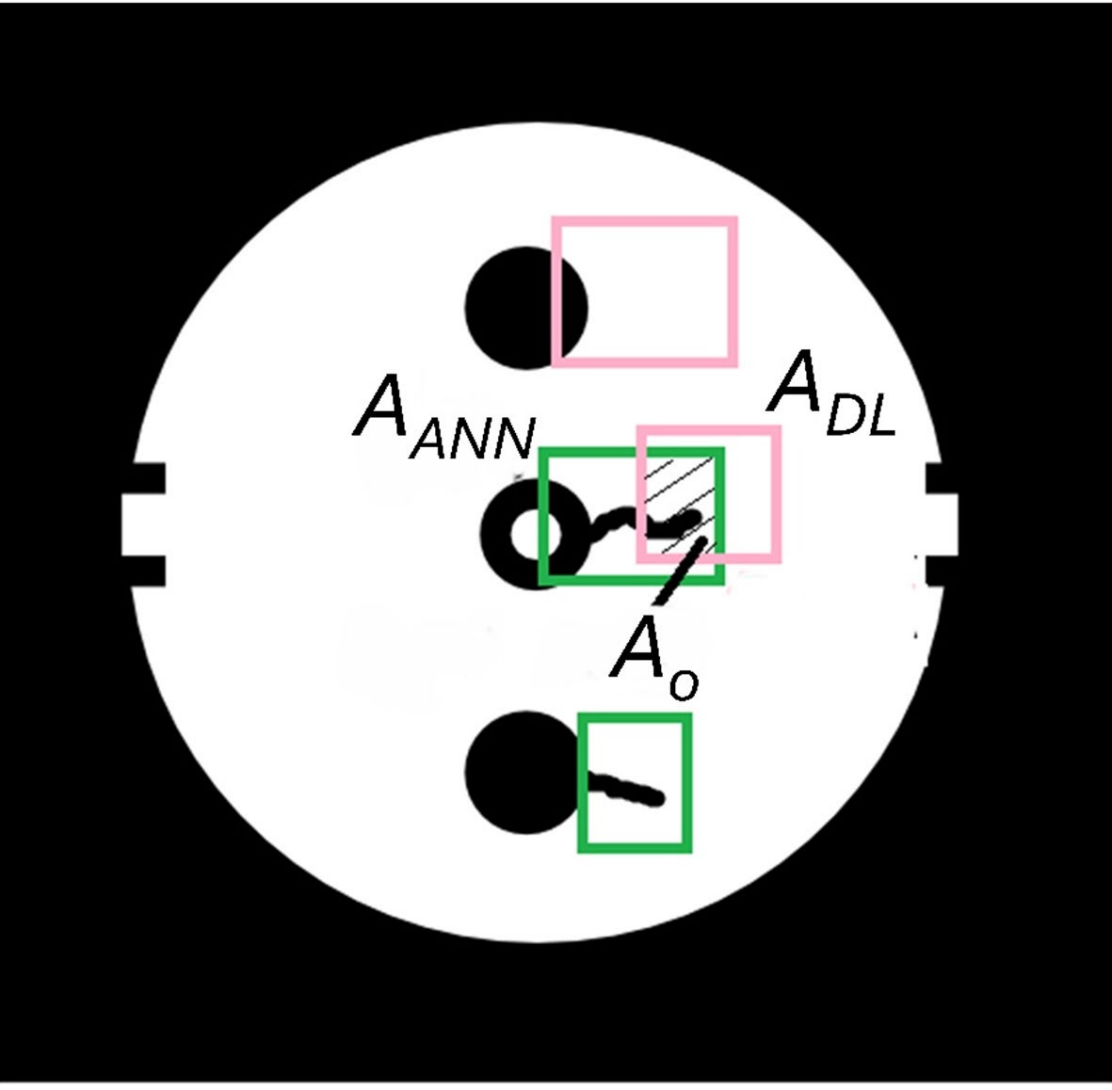
Visualization of bounding boxes: annotation labels (green) and predicted bounding boxes by the DL model (pink). The output of the object detection model is a list of bounding boxes. Ao and Au represent the overlapping area between each bounding box and the corresponding annotation label, and the union of the two, respectively.

To evaluate the detection results of the trained algorithms, different criteria from the literature, such as the ones deployed in the work of Meister et al.^7^, were considered. For each input image, the following metrics were defined:

- *ngt*: the number of manually annotated ground truth defects.
- *nd*: the number of detected defects predicted by the object detection model.
- While the goal is to classify parts as good or defective, tracking the number of detected defects can help evaluate whether the system is sensitive enough to identify anomalies across various conditions, such as different camera angles or positions of the part. Moreover, data redundancy enhances the AOI system’s overall reliability, as the number of defects is computed for multiple frames of the same part.
- *ndt*: the number of correctly detected defects predicted by the object detection model.
- According to its definition: *\:nd>ndt>ngt*
- *fp_rate*: false positive rate, calculated as $\:{fp}_{rate}=\frac{nd-ndt}{nd}$.
- *nd* does not account for non-defective areas that are mistakenly identified as defects. A high *fp_rate* (low precision) could lead to unnecessary rejections and increased operational costs.
- *fn_rate*: false negative rate, calculated as $\:{fn}_{rate}=1-\frac{ndt}{ngt}$.
- *nd* does not account for defective areas that are not detected. A low false negative rate (high recall) indicates that defects do not go undetected, which is vital for maintaining quality standards in manufacturing processes. Depending on the application, one might want to minimize the number of parts misclassified as good or defective, even though it is generally true that undetected anomalies cost the company more than incorrectly rejected good parts^40–42^.
- *IoU*: Intersection over Union for each detected defect, calculated as: $\:IoU=1-\frac{{A}_{o}}{{A}_{u}}$, where *Ao* and *Au* are the overlapping and union areas of two detection boxes, respectively.
- This metric is widely used (especially in segmentation tasks) to evaluate the accuracy of bounding box localization. A more comprehensive explanation of these concepts is provided with the use of Fig. 4.

For all inspection modes, predicted bounding boxes were retained using a confidence threshold of 0.25. During inference evaluation, a predicted defect was considered correctly matched to a ground-truth annotation when IoU ≥ 0.30. For part-level aggregation, K was fixed a priori to K = 3 and kept constant across all inspection modes.

Finally, the trained algorithms were tested using the data retrieved from the inspection videos of the testing samples. While a common practice involves collecting images of all available samples and then randomly splitting the dataset, this work kept the testing samples separate from the rest of the dataset. In practice, this approach involves first assigning the samples (manufactured parts) to either the training or testing set. Consequently, all frames captured from the videos of these parts are also assigned to the corresponding sets. As mentioned in the Introduction section, this method differs from many studies in the literature, where frames from all parts are initially collected and then randomly distributed across the sets.

The test set was formed by random sampling from the available production pool under a coverage constraint across defect-load levels (defined as the number of ground-truth instances per part). By construction, the resulting set is representative of the population of samples used in this work.

Inspection videos of another 26 samples (16 defective and 10 normal) were acquired for each technique. A certain number of frames was retrieved from each video, compatible with the average inference speed of the algorithm. The testing images were also annotated to evaluate the detection results.

Performance was evaluated at the part level (one statistical unit per physical part). To quantify uncertainty with limited test size (*N* = 26), 95% confidence intervals for part-level metrics (Recall and F1) were computed via non-parametric bootstrap resampling over parts (percentile method).

### Inspection Trajectory Optimization

In the first phase of the methodology, different techniques were compared based on the detection results obtained from videos where a generic inspection trajectory was followed. This strategy allows for a comprehensive inspection analysis of the part. However, some inspection time may be used to cover angles from which the defects are barely visible, leading to a low defect detection success rate. After conducting the inspection with a generic trajectory, its efficiency was evaluated to determine which camera angles led to the maximum detection rate. For a given total inspection time, the effectiveness of the inspection can be increased by focusing on angles that allow for a higher number of detected defects and giving less attention to “dead” angles. After the testing phase, a comprehensive analysis of the defect detection distribution along the trajectory was carried out. Based on the results, a new inspection strategy was defined, constrained to the same length as the original one. New samples were inspected with both strategies to assess the effectiveness of the optimization.

## Results and discussion

### Annotation phase

#### Inter-operator correspondence

Ground-truth labelling was assessed via one-to-one IoU matching at a 0.50 threshold and summarized by F1-0.50. The double-annotation yielded F1-0.50 ≈ 0.92, with 92% agreement on frame-level defect presence. All subsequent analyses employ the adjudicated consensus labels, and this correspondence level did not alter the comparative ranking of inspection modes.

#### Ground – truth labelling

Although the annotation phase of the dataset is a fundamental part of the development of a deep learning (DL) inspection tool, it is generally not included in the results discussion.

**Fig. 5:**
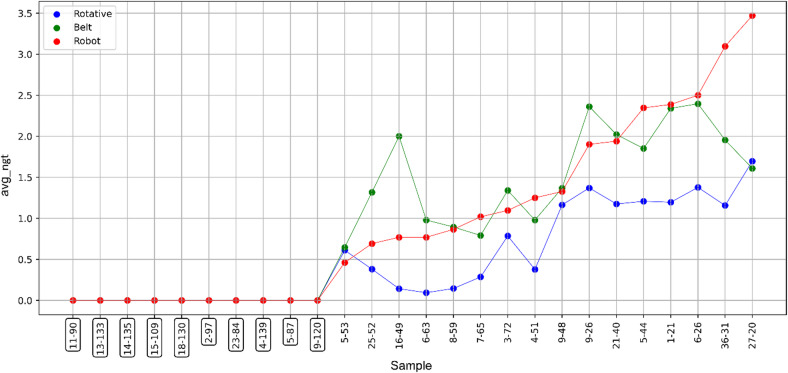
Average number of labelled defects per sample for different images acquisition techniques.

However, this work aims at comparing different inspection strategies that involve both data acquisition and data inference. Data collected for training the detection algorithms differ based on the chosen inspection mode, inevitably affecting the annotation phase. For each inspection mode, the number of annotated defects per image was counted for all retrieved frames related to the same sample. Since the total number of frames might change for each sample due to slight differences in raw video length, the average number of defects per image (*avg_ngt*) was calculated. Figure 5 shows the average number of defects per image for each sample under different inspection modes.

**Fig. 6:**
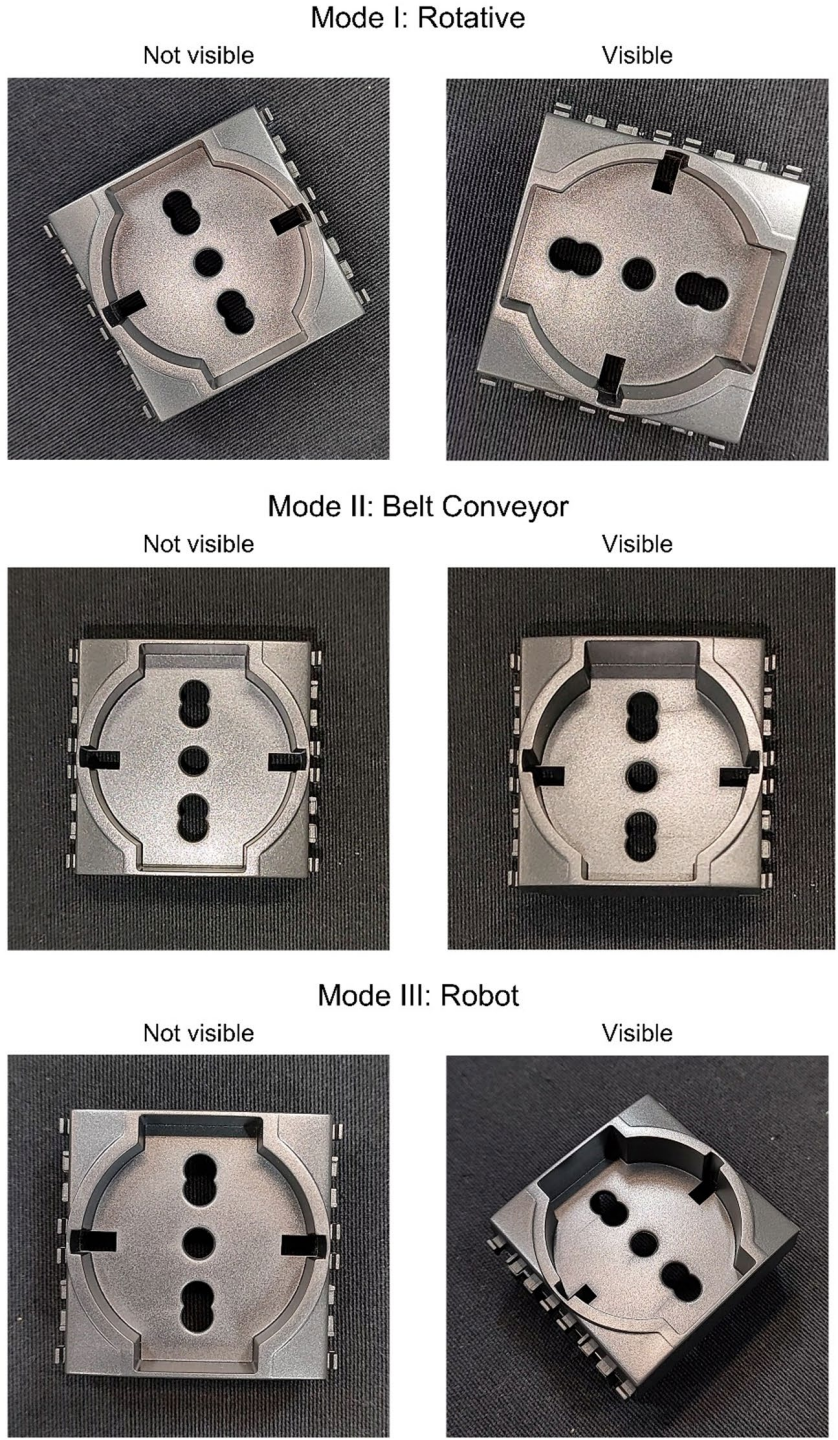
Comparison of defect visibility for different inspection modes for a reference sample (Part 1–21).

Each sample is assigned two numbers: the first number corresponds to its numbering within a specific category (defective, mildly defective, or normal), while the second number represents its absolute numbering across all samples. This dual numbering system helps distinguish between the sample’s position within its category and its overall position in the dataset. The framed labels on the x-axis correspond to the normal samples, while the others relate to defective samples. The data are arranged according to the increasing average number of annotated defects for Mode I. Considering only the defective samples, an increasing trend in the average number of annotations per image is observed for all three inspection modes. Despite the weld-lines always appearing in the same position and number due to the systematic behavior of the injection molding (IM) process, the extent of the defects can vary significantly from sample to sample. Moreover, the visibility of defects significantly depends on the inspection mode, with Mode I showing the lowest *avg_ngt*, while Mode II and Mode III show comparable results. Moreover, within the same inspection mode, different light reflections may either enhance the defect or cause it to nearly disappear. This inevitably affects the ability of the inspector to spot them while annotating the dataset. An example is reported in Fig. 6, which shows different frames of a defective sample for each inspection mode. Despite the images belonging to the same inspection video, it is clearly noticeable that some perspectives enhance defect visibility, while others make it almost impossible to detect. From a practical point of view, different images of the same part will thus be labeled either as defective or normal when annotating the dataset.

### Detection performance

For the detection results, the inference time of the algorithm was first considered. For YOLO-v8s, using 1024 × 1024 p images, the inference time was around 70 ms, making the detection algorithm suitable for this manufacturing context. Frames from the videos of the testing samples were sampled with a time interval of 2 s, resulting in 50 frames per sample.

**Fig. 7:**
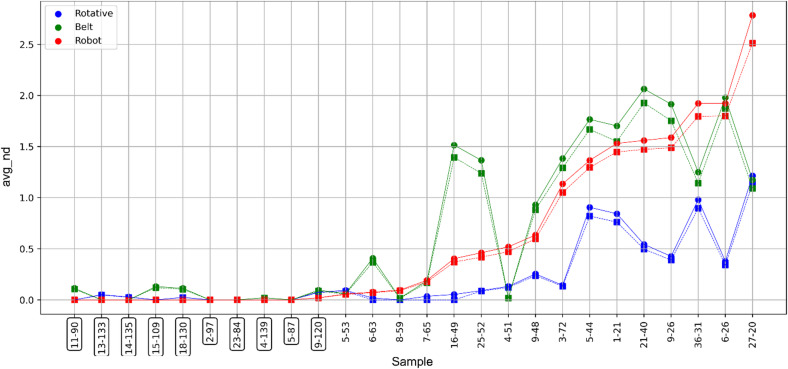
Average number of detected defects per sample for different inspection techniques. Round-shaped markers: nd, square-shaped markers: ndt.

The inference results on the testing dataset are shown in Fig. 7.

**Fig. 8:**
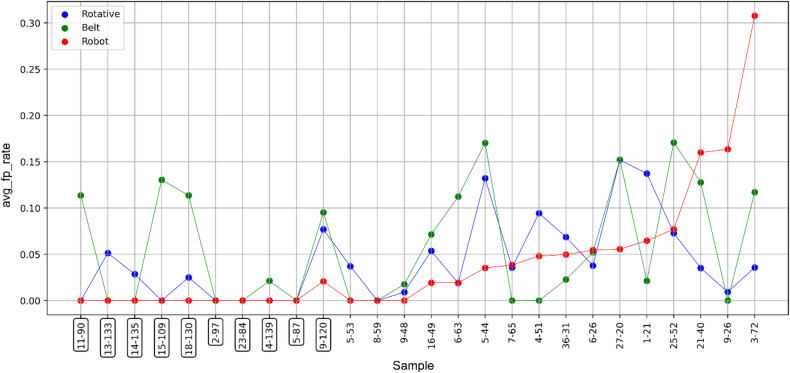
Average false positive rate per sample for different inspection techniques.

The data are arranged as before, according to the increasing average number of detected defects (*avg_nd*) for Mode I. Both the average number of defects per sample across all inspected frames (*avg_nd*), and the average number of correctly detected defects per sample (*avg_ndt*) are reported. While *avg_ndt* is useful for providing insights about the precision of the inspection method, *avg_nd* allows for an immediate understanding of potential erroneous detections when considering the good samples, as *avg_nd* can assume values greater than zero even though no defects were labeled in the analyzed image. Considering *avg_ndt*, it is observed that Mode I exhibits a significantly lower average number of detected defects across all samples compared to Mode II and Mode III. This suggests that rotating the part around its axis is not effective in enhancing the visibility of the weld-lines. The performance of Mode II and Mode III are almost the same, with some outliers in Mode II data due to the belt conveyor inspection strategy. In this strategy, the part is observed from a stationary camera during transportation, and only the angle φ between the camera axis and the part’s position on the belt changes. Therefore, initial positioning can significantly affect the inspection results. Another important observation is the presence of *avg_nd* values different from zero for some normal samples, indicating false positives. Figure 8 shows the average false positive rate per sample (*avg_fp_rate*). A non-zero *fp_rate* means the algorithm detects defects not matched by any ground truth annotations, leading to the rejection of good parts. Despite similar *fp_rate* values across all three modes, Mode II performs the worst in terms of false positives, with several normal samples erroneously classified as defective. In contrast, Mode III shows better robustness against false positives, as it only misclassifies defective parts.

**Fig. 9:**
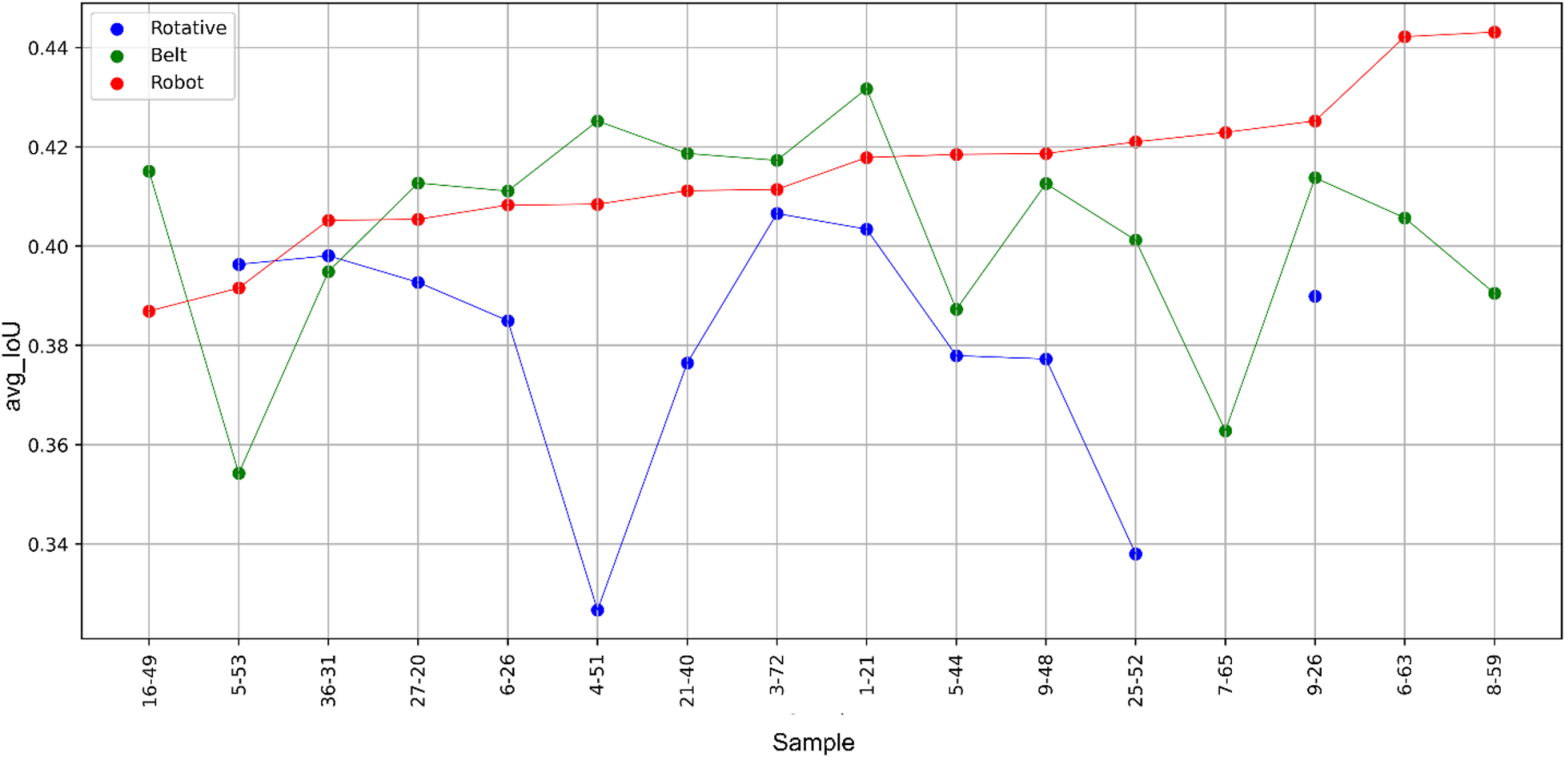
Average Intersection-over-Union for the defective samples. Missing data means that no defects were detected with that specific technique.

For the localization accuracy of the detected defects, the Intersection over Union metric (IoU) was considered. However, in our dataset the manually drawn bounding boxes are intended to simply enclose the defect region and their exact extent is inherently annotator-dependent (the same defect can be boxed slightly larger or smaller without affecting the inspection outcome). Therefore, very high IoU values are neither strictly expected nor required for the application, and IoU is treated here as a secondary indicator of localization consistency rather than a primary performance target. As shown in Fig. 9, the *IoU* ranges from 0.38 to 0.42 for almost all the samples. The IoU values indicate moderate localization consistency, which is acceptable for the present application because the inspection outcome depends primarily on detecting the presence of the weld-line defect rather than on highly accurate bounding-box localization. No significant differences can be observed between different modes, as the *IoU* is rather a characteristic of the detection algorithm itself, despite slightly lower results obtained with Mode I, which can be attributed to less accurate annotations. It is worth noting that for the inspection mode Mode I (Rotative), some data are missing (Sample 6–63, 7–65, 8–59, 16–49). This is due to the fact that for those samples, no defects were detected within these samples using this inspection method, thus no value of the *IoU* metric could be calculated. This is also confirmed by looking at Fig. 5, where, even though *avg_nd* ≠ 0, the value of *avg_ndt* = 0 for the above-mentioned samples.

The most crucial aspect of an AOI system is not missing any defective parts. In Mode II, some defective samples have both *avg_nd* and *avg_ndt* close to zero, indicating false negatives. These samples had defects that were only slightly visible but sufficient for the inspector to classify them as defective during annotation. Mode III, however, was able to recognize these defects in at least some frames, highlighting its effectiveness due to a wider range of inspection angles.

**Fig. 10:**
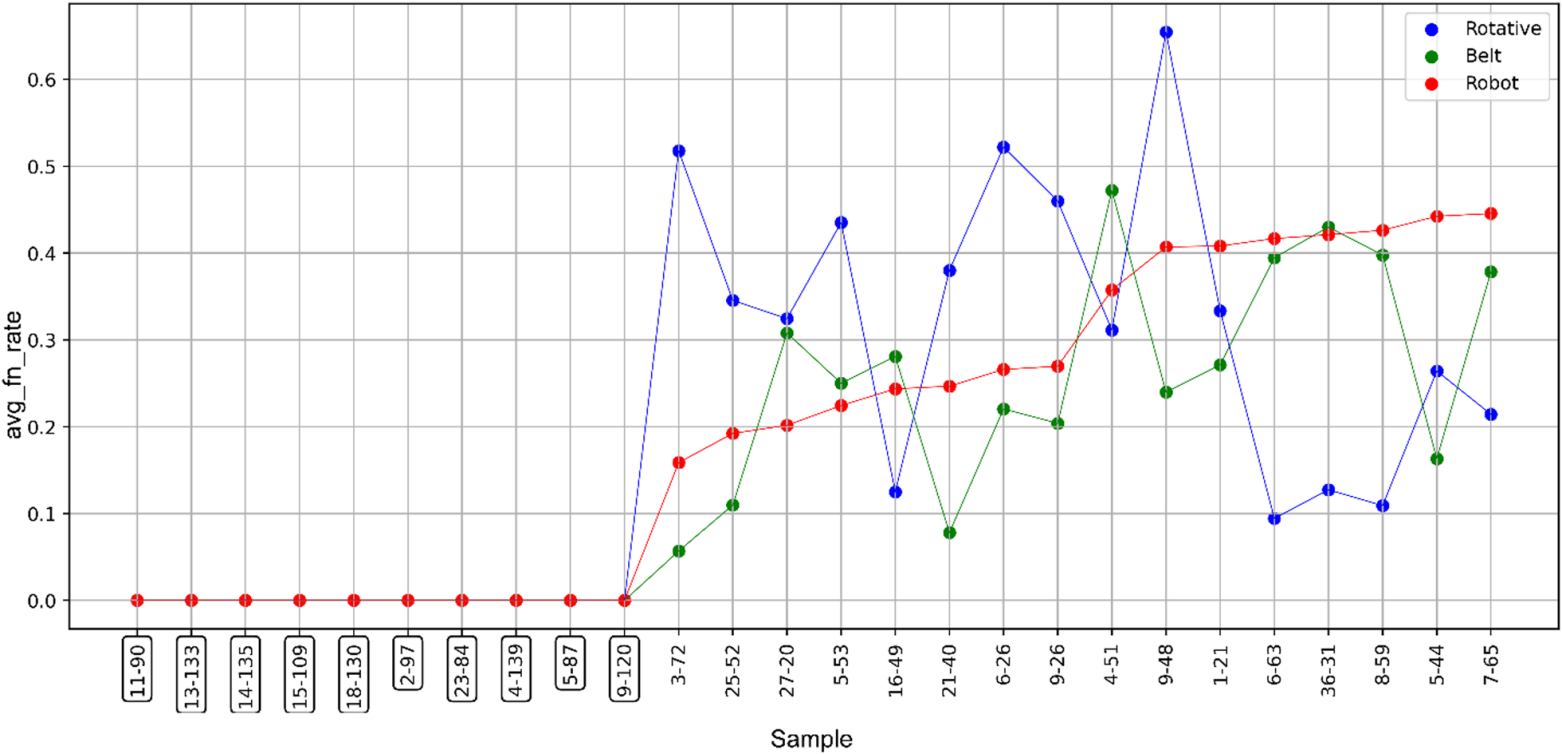
Average false negative rate per sample for different inspection techniques.

It is common practice to evaluate algorithm performance using the false negative rate (*fn_rate*) on the testing set. However, this approach may lead to erroneous conclusions. Figure 10, for example, might suggest similar detection capabilities for Mode II and Mode III. However, these results refer to specific frames, not sample. Despite comparable *avg_fn_rate* values, Mode III’s robot-based inspection strategy proved capable of detecting defects even in challenging scenarios where defects were only slightly visible.

**Fig. 11:**
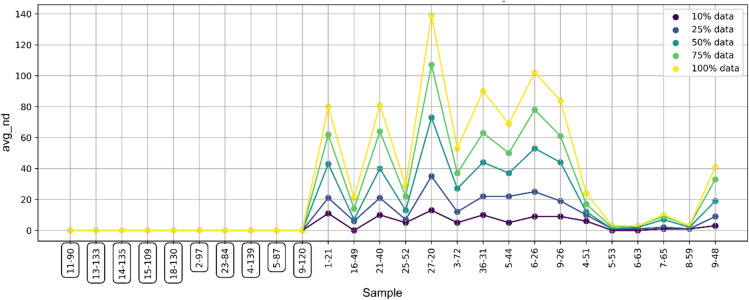
Average number of detected defects per sample for different percentages of frames used during the inspection.

Another crucial factor in quality control is the number of inspections per part. While it is a shared opinion that more inspections lead to more effective control, this aspect is often not deeply discussed. The maximum number of inspections depends on the production rate and the algorithm’s inference speed. In the IM process, cycle time can increase significantly with the part’s thickness due to longer cooling times, allowing for deeper inspection. In such cases, using an inspection video with varying observation angles can thus be more effective than static images. The available time and the algorithm’s inference speed determine the choice between static images and videos. Figure 11 shows the average number of detected defects (*avg_nd*) for different samples, varying the percentage of total frames used for inspection.

The increasing number of frames can be interpreted as a transition from static images to videos. As the number of inspection frames per sample increases, the number of detected defects rises almost linearly. Having multiple detection results also allows for a decision-making strategy to reject or accept the part based on a defect detection threshold rather than the results of a single frame.

The choice of the aggregation threshold K is context-specific and depends on the inspection protocol, in particular on the number of frames evaluated per part and on defect observability across views. The part-level good/defective decision is based only on predicted detections aggregated over the inspected frames, while matched detections are used only offline for performance evaluation against the annotated ground truth.

In a real production environment, the final part-level decision can only rely on the inference output of the detector, since ground-truth information is not available during inspection. Therefore, the good/defective classification must be based on predicted detections aggregated over the inspected frames, while matched detections are used only offline to evaluate the system performance against the annotated ground truth. No universal aggregation threshold can be defined, as the decision rule depends on the specific application and on the acceptable trade-off between false positives and false negatives. In many industrial contexts, rejecting a good part is preferable to allowing a defective part to pass inspection; therefore, even a single predicted detection may be used to trigger a warning or a conservative rejection decision. In this work, K was fixed a priori to K = 3 and held constant across setups to ensure a consistent part-level decision policy. A possible direction for future work is to consider a continuous notion of defect “visibility” across the inspected sequence, instead of fixed-threshold policies, to better adapt the decision rule to varying defect entity.

**Fig. 12:**
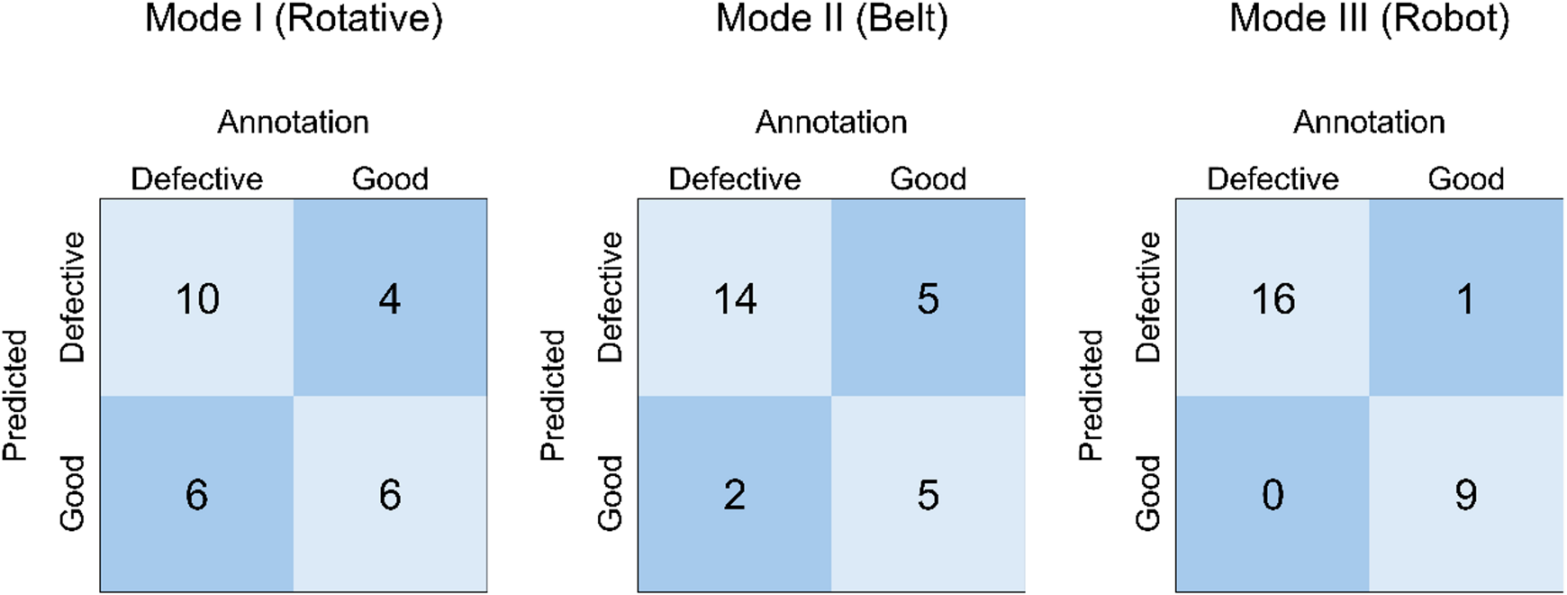
Confusion matrix for different inspection modes.

After completing the inspection of each sample, it is possible to determine whether the part should be classified as defective or normal. The analysis of the metrics discussed reveals that, while these evaluation metrics guide the decision to accept or reject a part, there is still room for additional considerations. For instance, even though the detection algorithm may identify defects in a specific frame, setting a minimum number of correctly identified defects for each complete inspection can help address the issue of false positives. Conversely, developing a strategy that focuses on increasing recall can significantly reduce the risk of failing to detect defective parts. In most manufacturing scenarios, it is generally more critical to prevent an anomaly from going undetected than to avoid rejecting a good part. However, this priority can vary depending on the specific circumstances (Fig. 12).

To complement the sample-by-sample analysis, Table 2 provides a comprehensive comparison across all inspected parts by summarizing the overall part-level performance of the three inspection modes in terms of Recall and F1-score, including 95% bootstrap confidence intervals as a measure of variability.

**Table 2:** Part-level performance on the test set. Values are point estimates with 95% bootstrap confidence intervals (resampling over parts).

Metric	Mode	Value	CI_LOW_	CI_HIGH_
Recall	Rotative	0.62	0.38	0.86
	Belt	0.88	0.69	1.00
	Robot	1.00	1.00	1.00
F1	Rotative	0.67	0.43	0.84
	Belt	0.80	0.62	0.92
	Robot	0.97	0.89	1.00

To quantify the uncertainty associated with the part-level confusion-matrix results, Table 2 reports non-parametric bootstrap 95% confidence intervals obtained by resampling the 26 test parts (the same physical parts were inspected under all three modes). On the full test set (Subset: ALL), Robot achieves the highest performance with Recall = 1.00 (95% CI [1.00, 1.00]) and F1 = 0.97 (95% CI [0.89, 1.00]). Belt shows intermediate performance (Recall = 0.88 [0.69, 1.00]; F1 = 0.80 [0.62, 0.92]), while Rotative yields the lowest values (Recall = 0.62 [0.38, 0.86]; F1 = 0.67 [0.43, 0.84]). Although the bootstrap interval is degenerate for Robot recall due to perfect detection on the available test set, this result should be interpreted within the limited sample size and validated on larger production datasets.

Overall, the confidence intervals indicate more robust part-level performance for Robot, whereas Belt and especially Rotative exhibit larger variability across resampled test sets.

These results quantitatively support the ranking previously observed at frame level. Mode I (Rotative) shows the weakest performance, with low precision due to numerous false positives and low recall indicated by many false negatives. In contrast, Mode II (Belt) improves in defect detection, with a noticeable decrease in undetected anomalies. Nonetheless, the best outcomes are achieved with Mode III (Robot), where there is a significant reduction in both undetected defects and wrongly rejected parts, confirming its effectiveness against both false positives and false negatives.

### Trajectory optimization

After analyzing the testing results, Mode III exhibited the best defect detection performance and the most robust inspection strategy against false positives. Despite its effectiveness, the adopted strategy is not the most efficient due to the generic camera trajectory used during inspection.

**Fig. 13:**
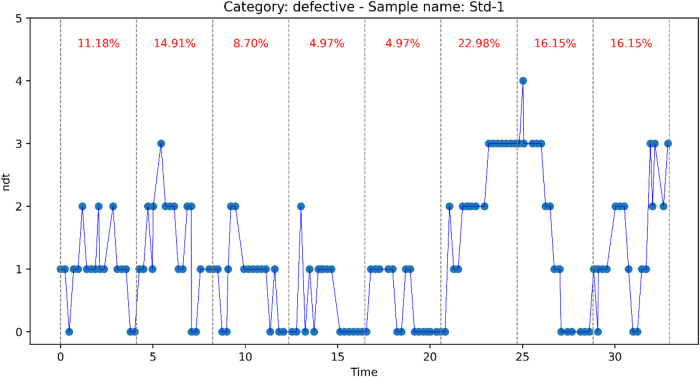
Number of correctly detected defects over time with the robot inspection technique for a specific sample.

Five new samples with varying defect visibility levels were inspected using Mode III. The detection algorithm was run directly on the inspection video, sampling frames at 200 ms intervals (5 frames per second). Figure 13 shows an example of the inference results for a specific sample.

**Fig. 14:**
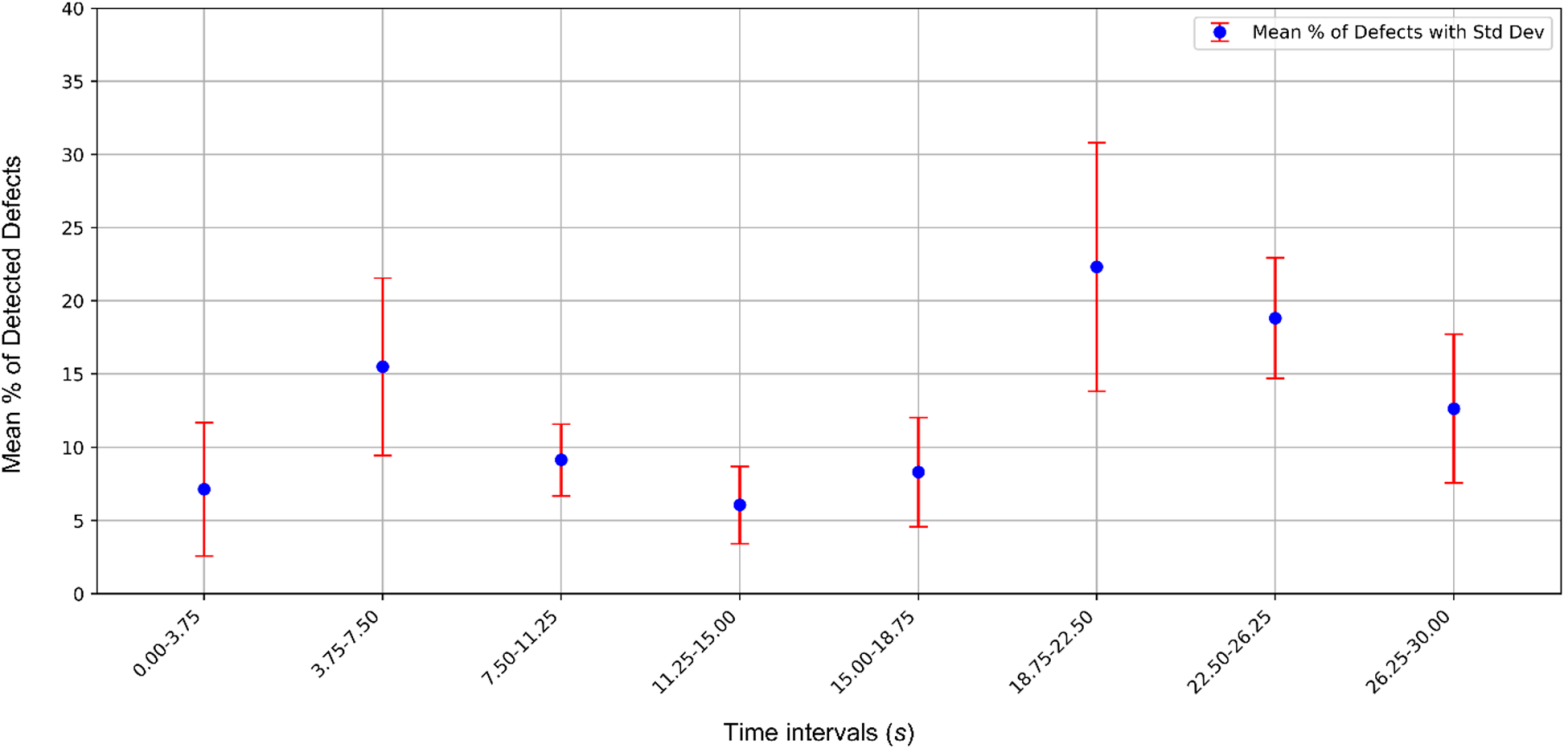
Mean percentage of detected defects per time interval among different samples.

The y-axis represents the number of detected defects per frame, while the x-axis shows time. The total video length (30 s) was divided into equal intervals, and the percentage of detected defects per interval was calculated. The percentage of detected defects varies with time, indicating specific camera angles that enhance defects visibility. These results are represented in an interval plot (Fig. 14), showing significant differences among group means, proving that different perspective angles can significantly affect defect visibility.

**Fig. 15:**
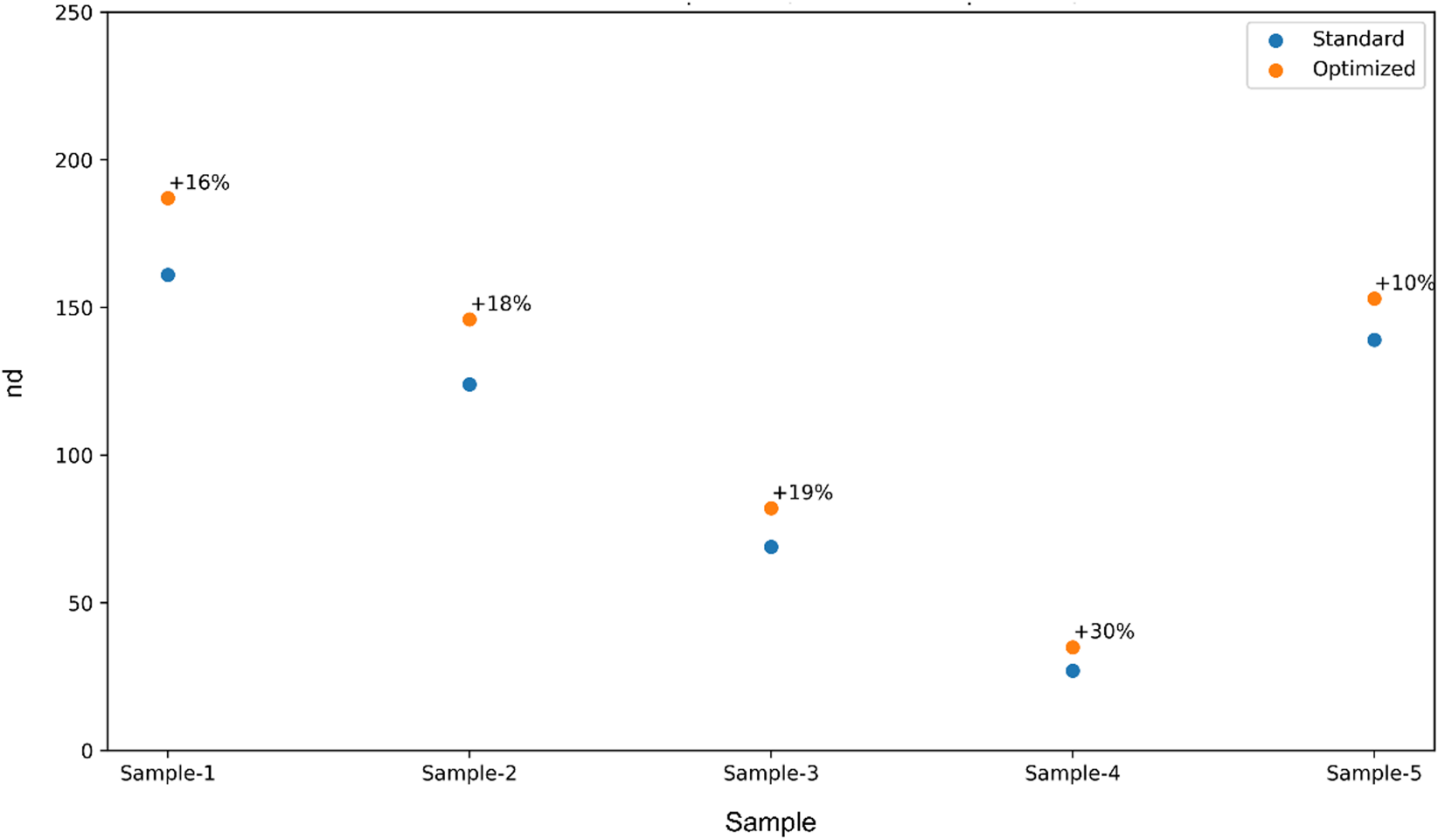
Number of detected defects with the robot inspection for different inspection trajectories.

Based on this analysis, the inspection strategy was optimized. The total inspection time remained fixed, but movements related to the lowest defect detection rate were minimized, and more time was spent on angles with the highest detection rates. Testing parts were then inspected using the optimized strategy, with results reported in Fig. 15.

The graph shows the total number of detected defects per sample for both the original and optimized strategies. The optimized strategy improved the defect detection success rate, with the percentage improvement noted for each sample.

The optimized trajectory was evaluated on a limited subset of five samples. Therefore, these results should be interpreted as preliminary proof-of-concept evidence of feasibility rather than as a statistically general conclusion. A broader evaluation on a larger and more diverse set of parts will be considered in future work.

## Conclusion

Due to the presence of hard-to-spot surface defects, product quality control of injection-molded (IM) plastic parts still requires considerable human intervention, especially during startups. This study compared three different AOI strategies for the quality control of a plastic cover affected by weld lines.

1. Mode I: Frontal inspection using a static camera with the part rotating around its symmetry axis.
2. Mode II: Belt conveyor inspection simulating part movement during transportation.
3. Mode III: Robot-based inspection allowing a wide range of camera angles around the stationary part.

Under these controlled conditions, the robot-assisted setup achieves the most favorable precision–recall trade-off, and a constrained trajectory time reallocation further increases correctly detected defective parts without increasing inspection time.

For each inspection mode, a dataset of annotated images generated from high-resolution videos was used to train the object detection model YOLOv8-s. The effectiveness of each inspection strategy was assessed based on inference results on testing samples with defects of varying visibility intensities, using custom metrics such as the average number of detected defects per sample, the false positive and false negative rates per sample, and the Intersection over Union. The effect of camera/part kinematics on AOI reliability is investigated by holding illumination constant and using mode-specific YOLOv8s models trained under the same architecture and hyperparameter settings across all setups. Decisions are made at the part level via a declared aggregation rule over multi-frame sequences. Mode I, exhibiting the highest false negative rate per sample, was the least effective inspection strategy, as it detected fewer defects per sample compared to Modes II and III. Mode II consistently identified the highest number of defects. However, it also resulted in the greatest number of false positives. Conversely, Mode III, despite detecting slightly fewer defects, showed better reliability in avoiding false positives. Moreover, Mode III was the most effective at identifying weld-lines in the most critical defective components, where defects were only slightly visible, thus indicating it as the most reliable technique under the tested conditions. An optimized inspection trajectory for the collaborative robot was then developed by analyzing defect detection distribution along different camera angles. The inspection conducted using the optimized path resulted in an 18% increase in defect detection. Additionally, the study examined the impact of using different numbers of frames for the inspection, comparing static images versus video sequences, to determine the most effective approach. The main contributions of this work to the field are:

- Proposing different inspection strategies to improve the detection success rate of hard-to-spot surface defects in IM parts, advancing the development of AOI systems in this field.
- Introducing a general methodology to evaluate and compare different setups of DL-based AOI systems. This encompasses the selection of raw data (static images or videos), determining the optimal number of inspection frames, comparing inspection strategies that allow modification of the observation angle, and optimizing the inspection strategy.

While the case study primarily focused on anomaly detection in injection-molded plastic parts, the proposed workflow is also applicable to other manufacturing fields that may experience complex surface disturbances leading to product rejection.

Although the experimental validation was conducted on a single component, the selected case study reflects conditions that are common across many injection-molded parts and are particularly challenging for AOI: a medium-size part with matte color, textured surface, and complex appearance variations under different viewpoints. Moreover, the considered defect type—weld lines—is often low-contrast and difficult to detect, making it a representative worst-case scenario for vision-based inspection. For these reasons, we expect the observed trends on the impact of acquisition setup (viewpoint/kinematics and multi-frame aggregation) to be relevant beyond the specific part analyzed, while quantitative performance values should be interpreted within the scope of the present dataset and test sample size.

Deploying an AOI system often requires time-consuming trial-and-error methods to compare different setup configurations, and this procedure could help in making decisions based on quantitative results. A critical parameter that requires substantial time to tune is the lighting, and the presented strategy could be used, for example, to evaluate and compare different positions and orientations of light sources within a single inspection mode.

Regarding the limitations of the proposed approach:

- Data Annotation: The effectiveness of the method significantly relies on the quality and comprehensiveness of data annotation. Variability in annotations can arise when different operators interpret the same dataset differently, potentially leading to inconsistencies where identical frames are classified alternately as defective or normal. Additionally, the same operator may classify the same dataset inconsistently at different times. To ensure accuracy, it is crucial to prevent biases, such as allowing operators to access information that links a frame to its corresponding sample.
- Short Cycle Times: In manufacturing processes with extremely short cycle times, the time available to capture and analyze images may be insufficient for thorough inspection. Under these circumstances, certain inspection modes, especially those that depend on complex camera trajectories, may not be feasible to implement. However, the proposed method can still be utilized to assess the impact of parameters that are available for modification.
- Hardware Cost: The cost of certain hardware required for inspection, such as collaborative robots, can be too expensive for some manufacturing setups. For this reason, while a robotic-assisted inspection mode may offer superior detection performance, its high cost may limit its feasibility for small-scale manufacturers or cost-sensitive production environments.

Overall, when comparing different setup configurations, it is crucial to first understand and define the operational and economic constraints. The proposed approach can then be utilized to evaluate the impact of one or multiple parameters on the effectiveness of the detection system.

### Actionable guidance for deployment

This subsection translates the identified limitations into concrete, operational measures for production use. For each constraint—annotation variability, cycle-time budgets, robot cost, false detections, missed defects, and illumination sensitivity—it outlines a corresponding mitigation and the decision criterion to apply. The goal is to support reproducible adoption while preserving high part-level recall under realistic manufacturing constraint.

- Annotation - Ground-truth variability.
  - Risk: borderline cases may yield inconsistent labels, affecting FP/FN at part level.
  - Mitigation: a small audit subset (e.g., 5–10% of the dataset) is re-annotated in double-blind fashion with adjudicated consensus; low-agreement cases are added to an “edge-case set” used for periodic audits and threshold checks.
- Cycle time constraints.
  - Risk: The available cycle time may not allow the frame budget that maximizes recall.
  - Mitigation: Frame sub-sampling (k-frame policy) is selected to meet a target part-level recall under a maximum cycle-time cap. The chosen setup is the one achieving the maximum recall given a maximum cycle-time.
- Robot cost.
  - Risk: Higher CAPEX/OPEX can hinder adoption.
  - Mitigation (cost rule): Setup selection is guided by expected per-part cost.

$$\:C={c}_{FN}{FN}_{rate\:}+\:{c}_{FP}{FP}_{rate}+c{T}_{cycle}$$

## Future work

This study examined a single injection-molded part. The comparative methodology can be extended to additional parts with similar optical and morphological characteristics. Further studies exploring different geometries and materials could enrich the evidence base.

## Data Availability

The datasets used and/or analysed during the current study are available from the corresponding author on reasonable request.
